# Development of a predictive model for PM_2.5_ over the greater Athens metropolitan area, Greece, at a 1 km by 1 km grid using satellite measurements and machine learning methods

**DOI:** 10.1371/journal.pone.0352975

**Published:** 2026-07-06

**Authors:** Seyong Chang, Evangelia Samoli, Evangelia Diapouli, Konstantina Dimakopoulou, Barrak Alahmad, Petros Koutrakis

**Affiliations:** 1 Department of Environmental Health, Harvard T. H. Chan School of Public Health, Boston, Massachusetts, United States of America; 2 Department of Hygiene, Epidemiology, and Medical Statistics, School of Medicine, National and Kapodistrian University of Athens, Athens, Greece; 3 Institute of Nuclear & Radiological Sciences and Technology, Energy & Safety, National Centre for Scientific Research “Demokritos”, Athens, Greece; 4 Department of Basic and Clinical Sciences, Medical School, University of Nicosia, UNIC Athens, Athens, Greece; Texas A&M University, UNITED STATES OF AMERICA

## Abstract

**Background:**

Ambient PM_2.5_ exposure is strongly associated with adverse health effects, including all-cause mortality. However, the lack of monitoring networks globally necessitates a better understanding of the spatiotemporal distribution of near-surface PM_2.5_ pollution. While ground-level pollutants are traditionally measured at fixed stations, integrating land use and atmospheric reanalysis data captures the broad geospatial trends and specific aerosol compositions necessary for high-resolution exposure assessment. This study aims to demonstrate the accuracy and reliability of ensemble modeling for estimating near-surface PM concentrations at a high spatiotemporal resolution in Athens, Greece.

**Methods:**

Daily PM_2.5_ concentrations were estimated using a stacked ensemble machine learning model to incorporate distributed random forest, gradient boosting, and feedforward neural network algorithms to minimize predictive error compared to individual models. The input set for all base learners consisted of daily observations from air quality monitors between 2007–2019 combined with satellite-derived estimates, providing a total of 61 variables describing regional aerosol, weather, and land use characteristics.

**Results:**

We observed strong predictive performance in our ensemble model, with a mean R^2^ of 0.85 and an average error of 4.18 μg/m^3^. The annual average concentration of PM_2.5_ (22.30 μg/m^3^) exceeded current WHO and EU guidelines, with considerable spatiotemporal variation across greater Athens. The highest annual mean PM_2.5_ concentrations were in 2007 (33.18 μg/m^3^) and on average year-to-year, PM_2.5_ concentrations were highest during the mid-winter months, in agreement with the expected seasonal maximum for the region, likely driven by increased residential heating alongside winter meteorological conditions, such as temperature inversions and a shallow, stable planetary boundary layer.

**Significance:**

This is among the first studies to estimate PM_2.5_ exposures in the greater Athens region at a high spatiotemporal resolution using diverse satellite and land use data. This framework enables the investigation of cumulative exposures, particularly in regions with limited ground-level monitoring.

## 1. Introduction

Air pollution is one of the leading environmental risks to human health worldwide [1,2]. Fine particulate matter (PM_2.5_), specifically, has been associated with numerous adverse short-term and long-term health outcomes, including cardiovascular disease, respiratory exacerbation, cancer, diabetes, morbidity, and mortality [3–8]. Recent studies suggest that PM_2.5_ not only penetrates deep into lung tissues and into the bloodstream, but may also be capable of crossing the blood-brain barrier, with emerging links to developmental delays and neurodegenerative disorders [9,10].

To better evaluate the health effects of PM_2.5_ exposure, environmental epidemiology studies require accurate, high-resolution estimates, often derived from air quality modeling, of ambient concentrations that study subjects are routinely exposed to in urban and rural environments. To date, several different estimation methods have been developed to assess particulate matter exposure in epidemiological studies, broadly categorized into physics-based chemical transport models and regression-based models. However, because PM_2.5_ concentrations are highly influenced by a complex interplay of emission sources, meteorological conditions, and topographical features [11–15], these traditional methods often face trade-offs that limits their generalizability across diverse geographical and study contexts.

Chemical transport models (CTM), including the Community Multiscale Air Quality Modeling System (CMAQ) and the Comprehensive Air Quality Model with Extensions (CAMx), are source-based models that can theoretically reproduce the spatiotemporal distribution of PM_2.5_ and its individual components based on emission inventories, meteorological data, and deterministic simulations of common atmospheric chemical and physical processes. While these models provide a comprehensive, process-based understanding of pollutant behavior in the atmosphere, they are often limited by their high computational demand and a reliance on the accuracy of the underlying source apportionment [16–19]. Furthermore, the typical spatial resolution of CTM can be too coarse to capture the local gradients found in urban environments, which can lead to significant exposure misclassification, particularly in regions where monitoring is sparse [20]. In contrast, regression-based models, including land use regression, geographically weighted regression, and mixed-effect models, are empirical approaches that can provide high-resolution estimates by combining air pollutant observations derived from limited monitoring locations and a variable amount of predictor variables. However, regression-based models are particularly susceptible to overfitting, may fail to capture the complex relationship between input variables when generating pollutant concentrations, and can potentially yield uninterpretable model coefficient estimates if several input variables are highly correlated against one another [21–24].

Consequently, a machine learning modeling framework can offer a viable alternative to generating high-resolution PM_2.5_ estimates for regions lacking dense, ground-level air quality monitoring infrastructure. Individual machine learning models have already been implemented to estimate PM_2.5_ in a diverse range of regional environments and spatiotemporal resolutions, including continental resolution for the contiguous United States, China, and Europe, regional resolution for Northern China, and city-level resolution for Mexico City [25–32]. However, because the model performance of a specific algorithm often varies significantly across different geospatial contexts, it is impractical for a single algorithm to optimally fit all regions. The most recent approach to reconciling the strengths and weaknesses of individual machine learning algorithms is to present a hybrid model. A stacked ensemble framework offers a superior alternative by reconciling these localized variances. Ensemble models can integrate multiple machine learning algorithms and leverage large datasets to improve predictive accuracy, capture complex relationships between pollutants and human-environmental factors when the underlying atmospheric dynamics can seem complex and elusive, and can be fit to multiple spatial and temporal resolutions without relying solely on predefined assumptions about atmospheric processes [33–36].

This versatility makes ensemble modeling particularly suitable for the Mediterranean basin, a region characterized by high environmental heterogeneity and complex pollution dynamics. Within this context, Southern Greece serves as a representative testing site for high-resolution ensemble modeling frameworks. To date, several studies have been conducted to quantify ambient PM levels in Greece and other Mediterranean countries [14,37–39]. These studies consistently report that annual PM levels in this region are closely linked to anthropogenic activity, especially from urban emissions and industrial processes. PM_2.5_ concentrations are also frequently observed higher than the limits set by both the World Health Organization (WHO) and the European Union (EU) Air Quality standards. In particular, Athens and its neighboring municipalities make up a densely populated metropolitan region of about 412 km², situated in a mountainous basin that traps air pollutants, with Piraeus to the south serving as both a major port and a key industrial and urban hub. Therefore, a localized, ensemble model is expected to achieve higher accuracy by better capturing intra-urban emission sources, meteorological patterns, and topographical influences [36].

In this paper, we propose the development of a daily PM_2.5_ estimation model of 1 km^2^ resolution across 13 years from 2007 to 2019 for PM_2.5_ over the greater Athens metropolitan area in Greece. By integrating public satellite-derived datasets on meteorology, aerosol composition, and land use into an ensemble machine learning framework, our model captures complex spatiotemporal variations in pollution levels without the need for a contiguous monitoring network. This approach provides a high-fidelity longitudinal dataset for public health research in Greece and establishes a scalable, transferable framework adaptable to urban regions globally that face challenges with limited monitoring networks.

## 2. Methods

Predictors for the proposed model were collected for Athens, Greece, between January 1st, 2007, through December 31st, 2019 (totaling 4,748 observation days). Our base models – distributed random forest, gradient boosting, and feedforward neural network – included a total of 61 predictors. These were broadly categorized into monitored pollutant concentrations from available air quality monitors (S1 Table), meteorological observations, satellite-observed aerosol data, and land use and cover characteristics (see S2 Table for individual predictors). Model inputs were selected to capture both spatial and temporal variability: spatially varying predictors, such as land use characteristics, characterize the physical and built environment that influences air pollution, while temporally varying predictors, like daily satellite-observations of aerosols, account for time-varying fluctuations in environmental conditions and pollutant levels.

To integrate predictors with differing spatial resolutions, we performed bilinear interpolation to resample coarse-resolution datasets (e.g., ERA5, MERRA-2) to our 1 km^2^ target grid. Only a single interpolation step was implemented to avoid compounding error propagation and to minimize artificial data smoothing. While further downscaling techniques exist, we opted for this approach to preserve the fidelity of the original environmental datasets and ensure that our model estimates remain grounded in the primary data, rather than in preprocessing artifacts.

### 2.1 PM_2.5_ monitoring data

We collected daily PM_2.5_ monitoring data from 15 fixed monitoring sites in Athens, Greece, maintained by the Greek Ministry of the Environment and Energy (GMEE) (S1 Fig). Although all air samplers operated continuously between 2007 and 2019, approximately 49% of the daily PM_2.5_ values were missing across the dataset, primarily due to intermittent gaps at specific monitors on certain days rather than complete network-wide outages. To broaden coverage and ensure sufficient response data for model training, we supplemented monitor observations with previously validated PM_2.5_ estimates (adjusted R^2^: 0.82) at selected sites (S1 Table) [40]. All monitoring sites with available observation days were ultimately included to maximize spatial coverage across the greater Athens metropolitan area and to allow the base machine learning models to better characterize fine-scale spatial heterogeneity in PM_2.5_ concentrations.

### 2.2 Aerosol optical depth data

We collected the mean aerosol optical depth (AOD, also interchangeable with aerosol optical thickness or AOT) data daily for Athens across the entire study time period at a spatial resolution of 1 km by 1 km, from the satellite remote sensing MODIS instrument, which is carried aboard the Terra, launched in December 1999, and Aqua, launched in May 2002, satellites of NASA’s Earth Observing System [41]. As circular sun-synchronous polar orbiting satellites, the Terra satellite has a local crossing time daily over Athens at approximately 10:30 and the Aqua satellite at approximately 13:30. Through the Collection 6 Multi-Angle Implementation of Atmospheric Correction (MAIAC) AOD dataset, AOD measurements can be derived from MODIS surface reflectance data [42]. The algorithm used in this collection improves upon traditional AOD retrieval methods by accounting for darker vegetation areas, brighter surfaces, and low-to-moderate cloud cover. According to the Earth Observing System’s global sinusoidal tile system, greater Athens contained approximately 5,226 grids.

We extracted AOD at 550 nm from both satellites using AOD quality control and validation methods and included all measurements that met the MODIS quality assurance criteria flag of “good” and excluded all measurements with quality assurance flags indicating those with high uncertainty, high cloud cover, and coverage over water bodies [25,43]. Due to technical factors like the relatively high surface reflectance in the region and low acquisition quality, approximately 43% of the total AOD data was missing. To address this, we estimated the linear relationship between the Aqua and Terra AOD values for each day and imputed missing AOD values when data from only one satellite were available [44]. If both satellites had missing data for a given day, a random forest model was developed to impute the missing values [45]. The final measurements used in the model were the average AOD at 550 nm from both Terra and Aqua satellites.

### 2.3 NO_2_ data

We collected daily NO_2_ concentrations in the study region from the 15 fixed monitoring sites run by the GMEE between 2007 and 2019. NO_2_ plays a significant role in the formation of particulate matter when reacting to other atmospheric compounds and its inclusion in the model is important to accurately estimate secondary particulate matter formation [46,47]. Although the NO_2_ data from the monitors represent ground-based measurements of concentration with units of µg/m^3^, satellite-based reanalysis and data assimilation products, such as the Sentinel–5P and GOME–2, commonly represent NO_2_ as total tropospheric vertical column densities with units of mol/m^2^. Because of the difficulties in converting from vertical column densities to ground-based concentrations, missing NO_2_ values (approximately 10%) from the air pollution monitors were imputed using the random forest algorithm [48].

### 2.4 Meteorological data – Monitored

We collected hourly meteorological data in the study region from a centrally placed weather monitoring station maintained by the National Observatory of Athens. All hourly data were averaged over a 24-hour period to match the daily resolution. The greater Athens metropolitan area is situated within a mountainous basin and experiences poor air-mass mixing, which has been shown to lead to more spatially homogenous weather conditions [49]. Therefore, meteorological variables were sourced from a single centrally located monitoring station, which likely represents daily meteorological conditions across the region. A total of 5 meteorological variables were measured, including recorded dust events (binary variable), mean 24-hour temperature (degrees Celsius), mean relative humidity at 1,000 hPa (%), mean wind speed (m/s), and a granular wind direction variable, dividing the full 360° into 17 compass-based directional zones.

### 2.5 Meteorological data – Satellite-derived

To account for additional meteorological influences on daily PM_2.5_ concentrations, we incorporated variables from the fifth generation European Centre for Medium-Range Weather Forecast atmospheric reanalysis (ERA5) [50]. As a global reanalysis product, ERA5 variables have been validated through comparisons with ground-based measurements [51]. We considered 8 meteorological variables selected to represent key atmospheric processes relevant to satellite retrieval, air pollution dispersion, and particulate formation, including planetary boundary layer height (vertical extent of atmospheric mixing), downward UV radiation at the surface (driver of photochemical reactions involved in secondary pollutant formation), evaporation, forecast albedo (the fraction of incoming shortwave solar radiation reflected by the surface), instantaneous 10m wind gust (turbulent transport and short-term dispersion events), surface pressure, total cloud cover, and total precipitation (sink process for atmospheric particulates through wet deposition). The variables were extracted at a spatial resolution of 0.25° × 0.25° (approximately 31 km^2^) and temporally aggregated from hourly to daily values.

### 2.6 Aerosol data

Because Southern Greece frequently experiences intense dust events originating from the Sahara and Arabian Peninsula Deserts, wildfire smoke, heavy traffic smog, and saline aerosols from the Saronikos Gulf, we included a wide range of surface– and tropospheric–level aerosol measurements to better characterize PM_2.5_ composition and variability. These included parameters describing mineral dust, organic dust, SO_4_ emissions, black carbon, and mixed aerosols (S2 Table). The data were sourced from the Modern-Era Retrospective analysis for Research and Applications version 2 (MERRA2), which is an atmospheric reanalysis platform provided by NASA for the approximation of surface-based climate observations at a spatial resolution of 0.625° × 0.5° (approximately 62.5 km by 50 km) and a daily temporal resolution [52]. MERRA2 data were available for the entire study period, spanning from 2007 to 2019. Previous studies have validated the reanalysis with the results of ground-based measurements and other satellite observations [53–55].

### 2.7 Visibility data

We calculated mean daily visibility from the available hourly visibility data for the greater Athens region. The relationship between visibility and PM_2.5_ is relatively consistent, as particles similar in size to visible light wavelengths scatter and absorb light—an effect especially pronounced in arid regions [56]. Visibility has also been used in prior studies to help predict historical ground-level PM_2.5_ concentrations in China before an air pollution monitoring network was established [57]. The hourly data was provided by the National Oceanic and Atmospheric Administration’s Integrated Surface Database, a data platform combining routine data from weather and research stations globally [58]. In Athens, the closest and only continuously operating station reporting visibility data was located at the Eleftherios Venizelos International Airport (station ID: 16716199999), located approximately 20 km east of the city center (see S1 Fig). The feasibility of using visibility data derived from airport-based monitors has been previously validated in locations in Southwest Asia [59], and Middle East [60].

### 2.8 Land use and land cover data

Various land use and land cover parameters were included as predictors in the model, including normalized difference vegetation index (NDVI), elevation, road density, and urban land use classifications. The NDVI data were obtained from the National Oceanic and Atmospheric Administration’s Climate Data Record, Version 5, through the Advanced Very High-Resolution Radiometer sensor, with a spatial resolution of 0.05° × 0.05° (approximately 5 km^2^) and a daily temporal resolution [61]. The elevation in each grid cell was obtained from the US Geological Survey’s Earth Resources, Observation, and Science Global 30 Arc-Second Digital Elevation Model (GTOPO30 DEM), which has a spatial resolution of 30 arc seconds (approximately 1 km by 1 km) [62]. Since traffic-related air pollution is a key contributor to urban PM_2.5_ concentrations, the total road length for each 1 km by 1 km grid was computed using the Overpass API to retrieve data from OpenStreetMap [63]. We also obtained the total area of 12 urban land use classifications for each grid cell across greater Athens. The urban land use classifications were sourced from the Copernicus Land Monitoring Service Urban Atlas for 2012 and 2018, with a minimum mapping unit of 0.25 hectares [64,65].

### 2.9. Missing data

Among the predictor variables, non-random missingness can occur due to several factors: (1) Some measurements were missing depending on where they were sampled from, such as AOD measurements over high cloud cover, water bodies, or high soil moisture areas. (2) Some measurements were censored from the training dataset during data processing due to predefined QA flags or high uncertainty, such as low-quality retrieval confidence or AOD measurements greater than 1.5. To prepare the training dataset for model prediction, robust methods to impute or fill in the missing values must be considered.

Where missing data accounted for a small proportion of the dataset (less than 10%) and measurements were assumed to change gradually over time, linear interpolation was used to estimate missing values. For example, this method was applied to fill gaps in meteorological variables, such as mean relative humidity and wind speed, by interpolating between the nearest observed temporal data points. When there were a large proportion of values missing, we trained separate prediction models using basic random forest algorithms, tuned using a grid search, to fill in and impute missing values based on non-missing predictors before ensemble model training and prediction. We applied this method of separate prediction models to fill in missing values for NO_2_ concentration and AOD at 550 nm (if neither satellite provided data), using meteorological data, aerosol composition, and land-use variables.

### 2.10 Base learners and ensemble model

We applied three base machine learning algorithms (also referred to as base learners) – distributed random forest (RF), gradient boosting (GB), and feed forward neural network (NN) – to model the complex relationship between input variables (also known as predictors) and the dependent variable (PM_2.5_).

Neural network is a deep learning algorithm based on a multi-layer feedforward artificial neural network trained using stochastic gradient descent through back-propagation [66]. Neural networks, with their ability to model complex and non-linear relationships, offer flexibility in capturing intricate patterns in the data, which helps reduce both bias and variance. In contrast, both random forest and gradient boosting are examples of algorithms that fundamentally utilize a series of decision trees [67]. In the random forest algorithm, decision trees are created independently and in parallel with random subsets of the training data, a technique known as bagging. Random forests reduce the model’s sensitivity to minor variations in the training set, improving the prediction variance. The final output is calculated as the average of the predictions from each tree. Gradient boosting, as a forward learning ensemble method, sequentially builds decision trees from each tree by optimizing a loss function to minimize residual error, a technique known as boosting. Gradient boosting reduces the difference between the expected prediction of the model and the true value of the target variable, iteratively refining the model’s predictions. The final output is calculated as the weighted sum of the predictions from each tree.

Finally, PM_2.5_ predictions from each base learner were fed into a higher-level generalized linear model to generate the final estimates and further improve overall performance. Because of the high likelihood that the estimates from each base learner are highly correlated, the regression coefficient weights were constrained to non-negative values. This allows each learner to contribute additively to the ensemble product, rather than offsetting the contributions of other learners through negative weights. This supervised ensemble technique, often known as stacking, attempts to identify the optimal combination of multiple prediction model outputs through a higher-level model to improve accuracy and generalization. The successful application of all three algorithms in a stacked ensemble approach to predict PM_2.5_ concentrations has been validated and compared against real-world data in previous air quality studies [56,68].

### 2.11 Model prediction, validation, and performance

**Fig 1:**
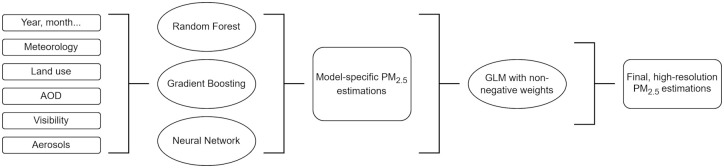
The flowchart of the model training process.

After imputing missing values and interpolating, complete input variables were available across the study area. We trained the three base learners and the ensemble model using these variables with monitored PM_2.5_ as the response variable and subsequently applied the trained models to the predictors at each 1 km × 1 km grid cell (n = 688 grid cells) to estimate daily ground-level PM_2.5_ concentrations from 2007 to 2019 in Greater Athens (Fig 1). To improve model accuracy and generalizability, we optimized hyperparameters for each base learner and the meta-learner using grid search, systematically evaluating combinations of parameters to identify those that minimized prediction error (see S3 Table for final model hyperparameters) [56,68–70].

The performance of all models was validated using 10-fold cross-validation (CV) of random 90% to 10% splits to avoid overfitting, where 90% of the training data was used for model training and the remaining 10% of the data was reserved for testing the model’s predictions. This process was then repeated 10 times to generate cross-validated model performance metrics, such as CV-R^2^ and CV-RMSE.

Following the aggregation of PM_2.5_ predictions and cross-validation results from all ten splits, we further assessed the full ensemble model for bias within-sites by calculating the temporal R^2^ and between-sites by calculating the spatial R^2^. The yearly temporal, spatial, and overall R^2^ values for the ensemble model were calculated as shown below:


$$\begin{array}{c} \overset{\text{2}}{\underset{temporal,y}{R}}=corr{\left(\Delta {PM}_{i,t,y},\Delta {\hat{PM}}_{i,t,y}\right)}^{\text{2}} \end{array}$$

$$\begin{array}{c} \overset{\text{2}}{\underset{spatial,y}{R}}=corr{\left(\overset{―}{{PM}_{i,y}},\overset{―}{{\hat{PM}}_{i,y}}\right)}^{\text{2}} \end{array}$$

$$\begin{array}{c} \overset{\text{2}}{\underset{overall,y}{R}}=corr{\left({PM}_{i,t,y},{\hat{PM}}_{i,t,y}\right)}^{\text{2}} \end{array}$$

Where: ${PM}_{i,t,y}$ is the observed PM_2.5_ concentration at a given monitoring site *i*, observation day *t*, and year *y*; ${\hat{PM}}_{i,t,y}$ is the model-predicted PM_2.5_ concentra*t*ion at a given monitoring site *i*, observation day *t*, and year *y*; $\overset{―}{{PM}_{i,y}}$ is the annual mean observed PM_2.5_ at site *i* and year *y*; $\overset{―}{{\hat{PM}}_{i,y}}$ is the annual mean predicted PM_2.5_ at site *i* and year *y*; $\Delta {PM}_{i,t,y}$ is *t*he difference between the observed PM_2.5_ and the annual mean observed PM_2.5_ at site *i*, observation day *t*, and year *y*; $\Delta {\hat{PM}}_{i,t,y}$ is the difference between predicted PM_2.5_ and the annual mean predicted PM_2.5_ at site *i*, observation day *t*, and year *y*.

We also assessed the relative importance and Shapley values of each predictor in estimating PM_2.5_ concentrations in each individual model. Relative importance scores estimate the impact of predictors on overall model fit and summarize how much each predictor reduced overall model output error across all trees or how sensitive the model output is to changes in each input feature [71,72]. In contrast, Shapley values estimate the impact of predictors on each individual prediction by quantifying their marginal contribution as a coefficient [73].

Finally, to assess the impact of the supplemented PM_2.5_ estimates, we compared our primary model results against a secondary sensitivity model (results provided in S4 Table). The primary ensemble utilized the full dataset (original + supplemented monitoring data), while the sensitivity model was trained strictly on the original GMEE monitor observations. This comparison allowed us to evaluate whether the increased spatial coverage provided by supplementation significantly altered the model performance metrics that could influence the estimated PM_2.5_ gradients.

All programming was implemented in the R software version 4.5.1 and mapping was implemented in QGIS version 3.44.3. The datasets and code generated during the current study are available in the Zenodo repository at DOI: https://doi.org/10.5281/zenodo.17517946.

## 3. Results

**Fig 2:**
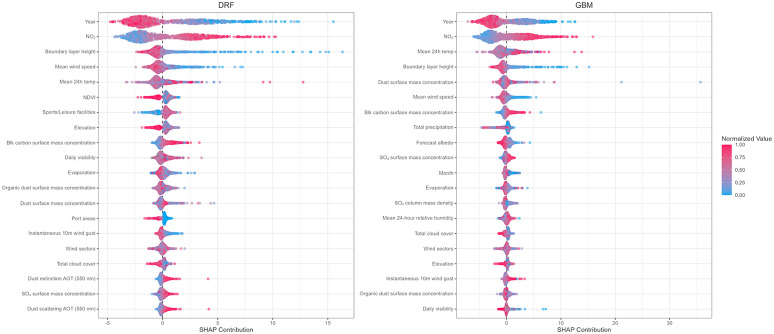
Comparison of SHAP-based feature contributions between the Random Forest and Gradient Boosting models. We calculated SHAP values for the top 20 features to quantify the relative importance and direction of estimation influence on modeled PM_2.5_ concentrations across the tree-based learners. Details of SHAP calculation and interpretation are described in the methods section.

We reported two measures of variable importance from the base learners. Table 1 summarizes the overall relative contributions across the base learners. For the random forest and gradient boosting models, variables such as NO_2_ concentration, date-related features, boundary layer height, and MERRA-2 total dust aerosols consistently ranked among the most influential to model fit. In contrast, the neural network model drew more heavily on wind direction, land use classifications, AOD-related variables, and MERRA-2 black carbon aerosols, suggesting that it contributed distinct, complementary information to the ensemble (Fig 2). These Shapley values reveal that although the features are ranked broadly in line with their overall relative contributions, there are notable differences between the RF and GB models in both the magnitude and direction of predictor effects on PM_2.5_ estimates, particularly at higher concentration levels. In general, the GB model assigned stronger positive PM_2.5_ contributions to several predictors, shifting estimates slightly further above the overall model baseline. For instance, higher NO_2_ concentrations in the GB model tended to push PM_2.5_ predictions further above the baseline compared to the RF model.

**Table 1:** The overall relative contribution of model features to each base learner model.

Random Forest	%	Gradient Boosting	%	Neural Network	%
NO_2_	15.16	NO_2_	22.72	All Wind Directions^*[b]*^	19.23
Date-related variables^*[a]*^	14.93	Date-related variables^*[a]*^	16.94	Land Use Classifications^*[d]*^	16.48
Boundary layer height	8.66	Boundary layer height	10.31	AOD-Related Variables^*[c]*^	10.68
AOD-related variables^*[c]*^	8.01	Mean 24-hour temperature	7.85	MERRA-2 black carbon aerosols	7.25
MERRA-2 total dust aerosols	6.37	MERRA-2 total dust aerosols	6.98	Date-related variables^*[a]*^	7.02
Land use classifications^*[d]*^	5.62	AOD-related variables^*[c]*^	4.30	MERRA-2 total dust aerosols	5.47
Mean 24-hour temperature	5.04	MERRA-2 black carbon aerosols	3.63	MERRA-2 SO_4_ aerosols	3.07
MERRA-2 black carbon aerosols	4.87	Mean wind speed	3.44	MERRA-2 organic dust aerosols	2.75
Mean wind speed	4.73	Land Use Classifications^*[d]*^	3.29	Mean 24-hour temperature	2.19
Daily visibility	3.06	All wind sectors^*[b]*^	3.04	Mean 24-hour relative humidity	1.87
All wind sectors^*[b]*^	2.28	Mean 24-hour relative humidity	1.86	NO_2_	1.77
MERRA-2 organic dust aerosols	2.22	Evaporation	1.84	Evaporation	1.73
Mean 24-hour relative humidity	2.18	MERRA-2 organic dust aerosols	1.68	Total precipitation	1.67
Elevation	1.88	Daily visibility	1.38	Latitude	1.66
NDVI	1.82	MERRA-2 SO_4_ aerosols	1.37	Downward UV radiation	1.54
Surface pressure	1.80	Surface pressure	1.25	Daily visibility	1.45
MERRA-2 SO_4_ aerosols	1.79	Total precipitation	1.19	Surface pressure	1.45
Instantaneous 10m wind gust	1.77	Instantaneous 10m wind gust	1.05	Forecast albedo	1.44
Evaporation	1.70	Total cloud cover	0.95	Total road length (all roads)	1.39
Downward UV radiation	1.25	Elevation	0.92	Boundary layer height	1.38

We calculated the relative contribution of each predictor variable for each learner based on the average decrease in model performance. Details of how relative contributions are calculated are described in the methods section.

^*[a]*^Includes year, month, day, and weekday.

^*[b]*^Includes all 16 precise wind directions.

^*[c]*^AOD-related variables include: AOD measured at 550 nm from the MAIAC-retrieved Terra and Aqua satellites and the extinction and scattering AODs of aerosol products from MERRA-2 (black carbon, SO_4_, organic dust, and total dust).

^*[d]*^Land use classification derived from Urban Atlas includes urban fabric levels, land used for railways, port areas, green urban spaces, industrial facilities, commercial facilities, public and private units, military facilities, and sports and leisure facilities.

The final ensemble model performed well overall, with strong performance across all model simulations (S4 Table) and within spatial and temporal domains (Table 2). The CV R^2^ values for the ensemble model ranged from 0.84 to 0.86, with a mean of 0.85 and a mean RMSE of 4.18 µg/m^3^. To ensure these results were not inflated by the use of supplemental PM_2.5_ data, we conducted a sensitivity analysis restricted to the directly monitored subset of the data. The performance metrics for this subset (S4 Table) remained highly consistent with our primary findings, demonstrating that our ensemble framework maintains its model performance even when evaluated exclusively against ground-truth measurements. This consistency is further evidenced by the ensemble model’s performance within spatial and temporal domains, where the spatial R^2^ ranged from 0.65 to 0.88, with a mean of 0.84, and the temporal R^2^ ranged from 0.66 to 0.89, with a mean of 0.79, supporting that our full ensemble model can capture the spatial and temporal variation of long-term PM_2.5_ relatively well. Both the spatial and temporal R^2^ showed a stabilizing trend in later years between 2015–2019 compared to the earlier study period, while the RMSE consistently declined. This suggests that as overall PM_2.5_ concentrations declined and stabilized, the model’s ability to capture consistent temporal patterns improved, likely due to a reduction in extreme pollution events that typically introduce higher variance. For the individual base learners, only the neural network learner underperformed relative to the other base learners, while the RF learner outperformed all other base learners in most years (Table 2). Despite the different algorithms applied, all of the base learners demonstrated good predictive performances, with mean R^2^ values of 0.823 for Random Forest, 0.843 for Gradient Boosting, and 0.799 for Neural Network (S4 Table). The overall ensemble model performance, however, exceeded the performance of any individual base learner.

**Table 2:** Cross-validated model R^2^ for the entire study area.

	Ensemble model						Random Forest	Gradient Boosting	Neural network
Year	R^2^	RMSE	Bias	Slope	Spatial R^2^	Temporal R^2^	R^2^	R^2^	R^2^
2007	0.84	5.85	2.09	0.96	0.85	0.78	0.849^*[a]*^	0.834	0.811
2008	0.86	4.68	0.87	1.00	0.85	0.74	0.849^*[a]*^	0.836	0.816
2009	0.84	5.06	1.88	0.96	0.88	0.66	0.845^*[a]*^	0.824	0.763
2010	0.87	5.29	3.39	0.87	0.65	0.69	0.812^*[a]*^	0.787	0.768
2011	0.83	4.85	1.77	0.95	0.81	0.78	0.826^*[a]*^	0.801	0.780
2012	0.85	4.78	2.34	0.89	0.85	0.76	0.817^*[a]*^	0.799	0.743
2013	0.87	4.85	0.82	0.96	0.85	0.69	0.800^*[a]*^	0.799	0.735
2014	0.84	4.48	0.50	0.98	0.87	0.77	0.825	0.826^*[a]*^	0.804
2015	0.86	4.38	0.08	1.00	0.82	0.89	0.861	0.861	0.837
2016	0.88	3.70	1.42	1.07	0.85	0.90	0.871^*[a]*^	0.865	0.848
2017	0.89	3.88	0.57	1.02	0.87	0.88	0.859^*[a]*^	0.846	0.806
2018	0.87	3.59	0.94	1.04	0.84	0.89	0.866^*[a]*^	0.859	0.819
2019	0.83	4.46	0.46	0.96	0.86	0.84	0.843	0.844^*[a]*^	0.754
**Overall**	**0.85**	**4.60**	**0.96**	**0.98**	**0.84**	**0.79**	**0.841**	**0.831**	**0.791**

All reported R² values were derived from 10-fold cross-validation of the ensemble model. Predictions were generated at the 1 km level using the trained ensemble. The methods for calculating daily, spatial, and temporal R² have been described previously [74]. To assess the slope and intercept (reported as bias in the table), we regressed predicted PM_2.5_ against monitored PM_2.5_ using a linear regression model.

^*[a]*^This learner outperformed all other base learners in that year.

**Fig 3:**
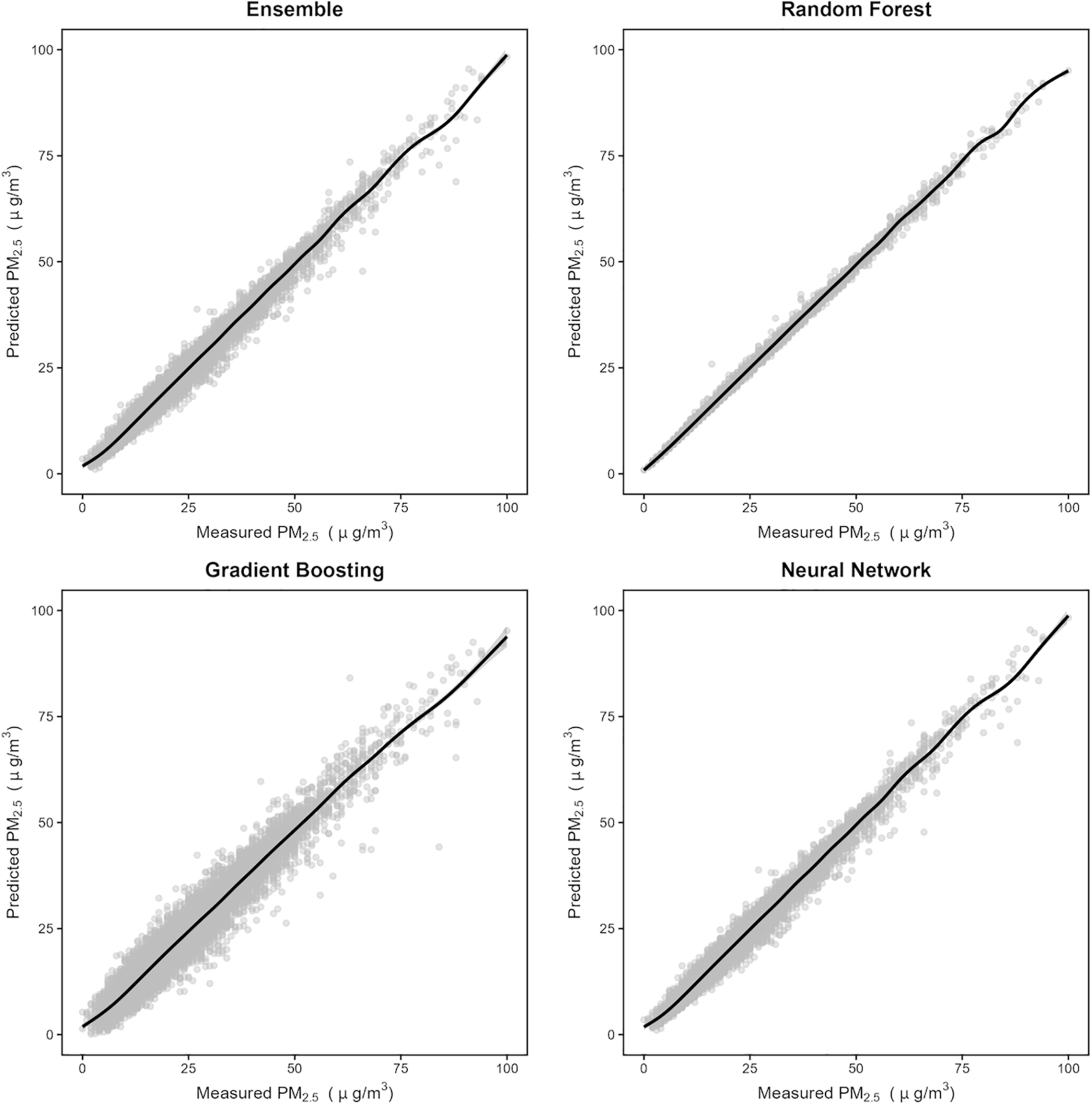
Relationship between monitored and predicted PM_2.5_ from the three base machine learning algorithms and the ensemble model. We regressed daily estimated PM_2.5_ from the ensemble model, random forest, gradient boosting, and neural network algorithms against monitored PM_2.5_ using a generalized additive model, with splines on the monitored PM_2.5_. The shaded regions represent the 95% confidence interval. All of the plots were bounded between 0 and 100 µg/m^3^, since 99.96% of daily PM_2.5_ estimates from 2007 to 2019 were below 100 µg/m^3^.

To quantify the added value of the stacking approach, we compared the ensemble performance against the best-performing individual base learner for each study year. On average, the ensemble framework yielded a mean marginal increase in R^2^ of 1.58% and a mean reduction in RMSE of 0.43 µg/m³. Because of the incremental increase in R^2^ from ensemble averaging over the best single learner, the improvement in the linearity of the relationship between measured and predicted PM_2.5_ was also relatively modest between the ensemble and individual base learners. For the ensemble model, the spline fit between measured and predicted PM_2.5_ remained fairly linear up to 75 µg/m^3^, a concentration rarely observed in the greater Athens region, indicating good performance even under conditions with limited monitoring data (Fig 3). The spline fits of the individual base learners showed varying performance. The ensemble model improved performance relative to the gradient boosting and neural network learners but slightly overestimated compared to the random forest, but whose fit suggested a slight-to-moderate degree of overfitting.

**Fig 4:**
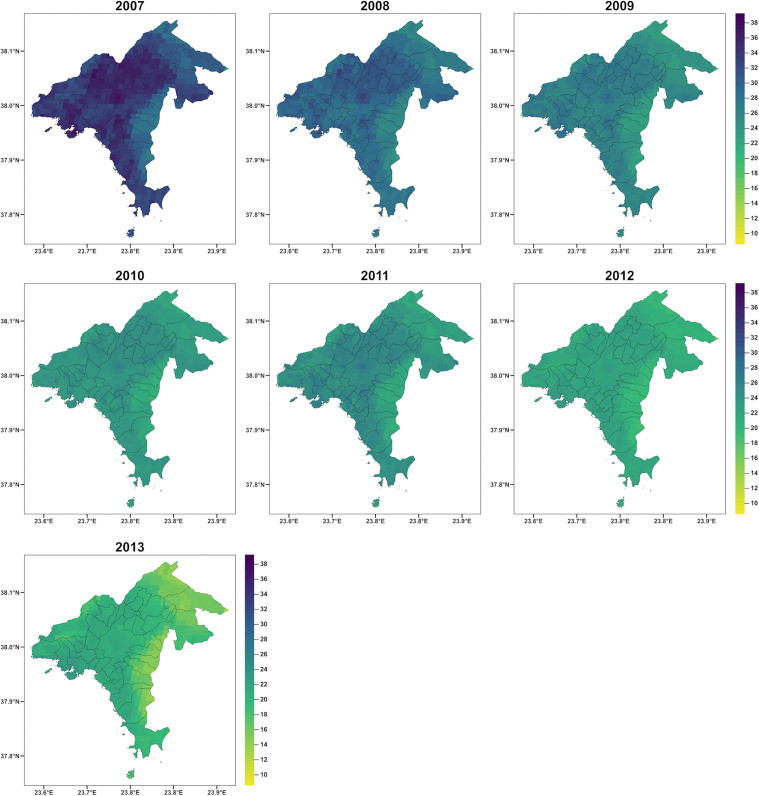
The spatial distribution of predicted annual PM_2.5_ concentrations, 2007 to 2013. Basemaps for the administrative municipality boundaries were obtained from the Athens GIS Repository [75]. We predicted PM_2.5_ concentrations for every 1 km by 1 km grid cell in the contiguous greater Athens region and then calculated annual averages per grid cell for the years 2007 to 2013. All maps were plotted at the same color scale, using the Greek Grid coordinate reference system (EPSG:2100; GGRS87).

**Fig 5:**
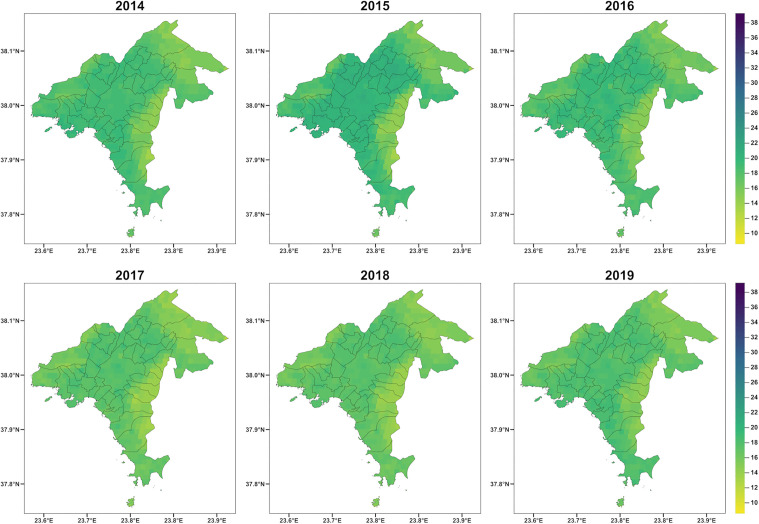
The spatial distribution of predicted annual PM_2.5_ concentrations, 2014 to 2019. Basemaps for the administrative municipality boundaries were obtained from the Athens GIS Repository [75]. We predicted PM_2.5_ concentrations for every 1 km by 1 km grid cell in the contiguous greater Athens region and then calculated annual averages per grid cell for the years 2014 to 2019. All maps were plotted at the same color scale, using the Greek Grid coordinate reference system (EPSG:2100; GGRS87).

Figs 4 and 5 map the spatial distribution of the predicted mean PM_2.5_ for each year. The mean predicted PM_2.5_ concentration for the greater Athens region across all years was 22.30 µg/m^3^, about twice the revised EU Ambient Air Quality Standards of 10 µg/m^3^. Overall, PM_2.5_ concentrations were moderately higher in the central, western and northwestern municipalities of greater Athens, where localized PM_2.5_ concentrations were approximately 5–8 μg/m^3^ higher than PM levels observed in the rest of the Athens metropolitan area and other urban regions of Greece (S2 Fig). Elevated predicted PM_2.5_ concentrations were observed in municipalities with high urban density (central Athens) and areas with significant industrial activity at the Port of Piraeus and the refineries of Elefsina, Aspropyrgos, Peristeri, and Aigaleo, municipalities where annual mean PM_2.5_ levels frequently exceeded 30 µg/m^3^. In contrast, more residential and mixed-use areas of the northern and eastern municipalities had annual mean PM_2.5_ concentrations generally at or below 20 µg/m^3^, although still above the fine particulate thresholds proposed in the revised EU Ambient Air Quality Standards.

**Fig 6:**
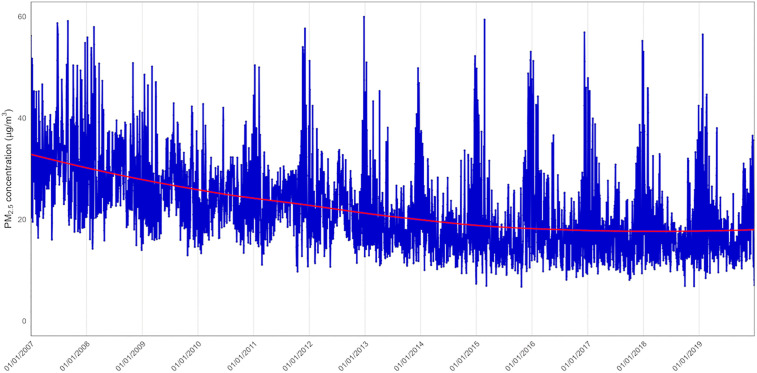
Aggregated daily PM_2.5_ concentrations (µg/m^3^) across greater Athens during the entire study period. We aggregated the daily municipality-wide averages (blue line) by averaging the daily estimations at all 1 km by 1 km grid cells. We then calculated the annual municipality wide averages by fitting a GAM-smoothed line (red line).

The highest annual PM_2.5_ concentrations were observed between 2007 and 2009, particularly in the western municipalities with major port and manufacturing activity, while the lowest annual PM_2.5_ concentrations were observed in 2014 and again from 2017 and 2019. In general, all municipalities had a consistent decline in annual mean PM_2.5_ levels across all years (S2 Fig). Seasonally, PM_2.5_ concentrations peaked during the months of November to February, which is consistent with seasonal variations in PM_2.5_ in Athens (Figs 6 and S3 describing the daily, monthly, and yearly trends of the modeled PM_2.5_ concentrations). Compliance with air quality standards also reflected this downward trend. In 2009, only six municipalities—Kessariani, Kifissia, Papagos-Cholargos, Penteli, Vyronas, and Zografos—met the then-current EU air pollution standard of 25 µg/m^3^, but by 2010, 31 municipalities fell within the standard. From 2012 onward, all municipalities remained in compliance, with PM_2.5_ concentrations below 25 µg/m^3^. However, under the revised 2024 EU standards, which lowered the annual PM_2.5_ limit to 10 µg/m^3^, no municipalities or subregions (Center, Piraeus, North, South, East, West) met the permissible PM_2.5_ concentration in any study year.

## 4. Discussion

We developed an ensemble machine learning model to estimate daily historical PM_2.5_ exposures at 1 km × 1 km resolution for the greater Athens metropolitan area, incorporating the limited monitoring system and publicly available satellite-derived data products. Our results indicated that this region had very high PM_2.5_ levels, which varied by location and year, and gradually declined year-over-year between 2007 and 2019.

The annual mean predicted PM_2.5_ concentration for the greater Athens area (22.3 µg/m^3^) from 2007 to 2019 was significantly higher than the newly revised annual air quality guidelines set by the WHO (5 μg/m^3^; in 2021), US National Ambient Air Quality Standard (9 μg/m^3^; in 2024), and the EU Ambient Air Quality Standards (10 μg/m^3^; in 2024) with certain years and regions exceeding these limits by more than two to fivefold. Few studies have measured PM_2.5_ exposures and modeled PM_2.5_ estimates in the greater Athens area and surrounding regions. Chalouakou et al. (2003) first recorded daily PM_2.5_ samples between June 1999 to May 2000 using a single, self-deployed low-volume air sampler in downtown Athens and later extended this line of research by analyzing daily PM_10_ samples between 1999 and 2001 from a central monitor in Athens and comparing the performance of neural networks versus multiple linear regression models in predicting PM_10_ concentrations [37,76]. Dimitriou et al. (2023) assessed hourly PM_2.5_ concentrations using 14 personal air quality sensors in 5 major Greek cities from 2019 to 2021, including 4 sensors in Athens [38]. In 2024, Dimitriou and colleagues expanded the analysis by using hourly PM_2.5_ concentrations from 2016 to 2022 using 11 public monitors across 6 major Greek cities, again including Athens [77].

Our estimates of PM_2.5_ seasonality in greater Athens also aligned with the seasonal trends of PM_2.5_ described in Dimitriou et al. (2024) and Siouti et al. (2024), who applied a chemical transport model in Patras, another Greek coastal city with similar built environmental characteristics [77,78]. Furthermore, the spatial distribution and range of PM_2.5_ estimates year to year are consistent with a similar study conducted by Kakouri et al. between 2015 and 2022 modeling high-resolution PM_2.5_ concentrations across Greece as well as with comparable European studies that estimate fine-scale air pollution levels using a broad variety of linear, algorithmic, and land use-based spatial modeling approaches [36,79,80]. However, their mean PM_2.5_ concentrations across greater Athens (8–15 µg/m^3^) were generally lower than our estimates, likely due to their inclusion of the years 2020–2022, a period during which a reduction in anthropogenic activity may have moderately reduced ambient pollution levels due to the COVID-19 pandemic [81–84].

Overall, the variability across the region indicated that residents living in greater Athens experienced fluctuations in personal PM_2.5_ exposure. Spatially, the central, western, and northwestern regions of greater Athens experienced the highest PM_2.5_ concentrations (Figs 4 and S2). While elevated PM_2.5_ concentrations in the west and northwest are primarily driven by industrial and maritime activity centered around the Port of Piraeus, the high concentrations in central Athens are largely a product of extreme urban density. This central core is characterized by high mixed-use developments and a lack of green spaces, with a per-capita density among the lowest in Europe, which limits the city’s capacity to disperse or mitigate local emissions [85]. These localized disparities illustrate how, at smaller regional spatial scales, PM_2.5_ levels are highly influenced by land use patterns, meteorological shifts, and near-road particulate contributions [86,87]. In addition to these spatial patterns, PM_2.5_ concentrations declined gradually across the entire region and in all sectors between 2007 and 2019 (Figs 4 and 5), which is likely due to a combination of severe economic recession from the Greek sovereign debt crisis, stricter European Union emission standards from the Euro 5 and 6 engines mandated for new vehicles (particularly for light commercial vehicles like taxi fleets), and stricter local emission controls. Daily (Fig 6) and monthly (S3 Fig) time series of PM_2.5_ concentrations from our model also highlight consistent peaks during the winter seasons, which could be attributed to normal temperature gradient inversions that trap local emissions such as residential biomass combustion, a home heating method that became popular during and after the Greek financial crisis [88].

Further differences in the PM_2.5_ concentration are reflected in the distinct variables prioritized by each base learner. The relative importance (Table 1) and Shapley values (Fig 2) of each predictor varied slightly between each base learner algorithm included in the ensemble model. Our random forest base learner depended heavily on NO_2_, followed by date-related variables, boundary layer height, AOD-related variables, and MERRA-2 total dust aerosols. Following a similar pattern, our gradient boosting base learner also depended heavily on NO_2_, followed by date-related variables, boundary layer height, mean 24-hour temperature, and MERRA-2 total dust aerosols. However, our neural network base learner was more nuanced, ranking all wind directions as the highest contributing predictor, followed by land use classifications, AOD-related variables, MERRA-2 black carbon aerosols, and date-related variables. The contribution of NO_2_ was rather negligible in the neural network algorithm (1.77%), suggesting that this base learner may have captured more complex non-linear relationships among other predictors to predict PM_2.5_. For example, the complex interactions between anthropogenic land use and meteorological conditions, both of which jointly affect PM_2.5_ formation and dispersion, may not be fully effectively understood by tree-based models but may be better captured by the flexible learning capacity and subtle feature sorting of neural networks. As a tradeoff, the neural network learner still showed reduced stability at higher PM_2.5_ concentrations above 75 µg/m^3^, likely reflecting its sensitivity in data-sparse regions of the distribution. This limitation is consistent with the general requirement of neural networks for large training sets to robustly capture patterns across the full available concentration range. In greater Athens, where extreme PM_2.5_ values are relatively uncommon, the lack of extreme data may have contributed to overfitting in the upper range, with the model most likely learning spurious patterns and becoming overly responsive to noise. That said, the ensemble model maintained a relatively stable linear trend within the reasonable range of PM_2.5_ concentrations measured in greater Athens, aligning more closely with the predictions of the tree-based learners. This suggests that the ensemble model potentially corrected any overfitting bias by the neural network base learner, resulting in more reliable predictions across both typical and extreme PM_2.5_ levels.

A limitation of our approach is that the final ensemble model did not substantially outperform the base models. This likely reflects the strong independent performance of each base model and their reliance on the same set of predictors, leaving limited extra information for the meta-learner to leverage by combining the model outputs. Future studies in the region should focus on incorporating more types of base models, especially non-tree-based models as well as incorporating a wider variety of data from different research groups and retrieval methods, including available chemical transport model data. Another limitation is that the AOD predictors in our model exhibited moderate non-random missingness, primarily driven by high cloud cover. Although the large proportion of missing AOD data could have introduced bias, the model accounted for this by incorporating missingness as a separate category and using model parameters customized by grid search to optimize performance across varying predictor patterns and data distributions, an approach that has been validated in previous studies [89,90]. Additionally, although computationally intensive, we fit a separate prediction model for AOD at 550 nm prior to model training and prediction to avoid data leakage. While some residual bias may remain, these steps help ensure that the model remains internally consistent and reliable for comparative inference. Finally, our use of standard k-fold cross-validation may potentially introduce spatiotemporal autocorrelation into the base models’ outputs by not fully isolating site-specific dependencies. However, it remains a computationally efficient method of evaluating overall model performance accepted in air quality modeling for historical exposure estimation, offering a balanced trade-off between bias and variance that is well-suited for our temporal scale [91–94]. Compared to other cross-validation approaches, which can be computationally prohibitive and susceptible to higher variance in data-sparse regions, our approach provides a stable validation metric that consistently demonstrates the ensemble’s ability to interpolate within our established monitoring network.

Our modeling framework offers a blueprint for others to replicate or adapt in similar geospatial settings. **First**, our model was developed for the local greater Athens area — Greece’s largest metropolitan region — and one that reflects many characteristics of other urban centers facing similar environmental and infrastructural challenges. Many comparable regions often lack even minimal air quality monitoring networks, highlighting the broader applicability and potential scalability of our modeling framework. In particular, this framework is well-suited for Mediterranean and North African contexts, where researchers continue to contend with the complex interplay of coastal meteorological influences and frequent Saharan dust events. Our approach’s modular design allows these regional stressors to be integrated as specific predictors alongside standard, publicly available satellite-derived data. While the core algorithmic architecture remains universal, the specific feature engineering can be calibrated to account for these unique local dynamics, providing a flexible, high-resolution solution for data-sparse environments. Our model results revealed persistently high PM_2.5_ levels across the region, with notable variation year by year and between municipalities, even as overall concentrations have gradually declined. **Second**, our model relies on a broad and diverse set of predictors, drawn primarily from publicly available global datasets. Many previous studies have chosen to focus on only AOD-PM_2.5_ or visibility-PM_2.5_ relationships to directly assess the spatiotemporal distribution of PM_2.5_, while our model highlights the importance of incorporating a diverse set of predictors, such as planetary boundary layer height, to better capture the complex processes influencing PM_2.5_ formation, accumulation, and dispersion. In this framework, fine-scale land-use predictors serve as the primary drivers of spatial variability, while conversely, coarse-resolution meteorological and aerosol fields provide the necessary atmospheric context, accounting for regional temporal dynamics that govern particulate dispersion and trapping. By leveraging these complementary data sources, our ensemble can remain high-resolution while accounting for both localized and broader contributors of PM_2.5_ that are often overlooked in simpler models. **Third**, we developed separate prediction models for NO_2_ and AOD predictors to help fill in non-random missingness. When the probability of a value being missing depends on the unobserved value itself, traditional statistical methods for handling missing data may distort seasonal trends or spatial patterns, introduce systematic bias into modeled estimates, and reduce model performance and generalizability in real-world applications. Previous studies have addressed missing data in various ways: censored observations or predictors with a significant amount of missing data, used multiple imputation by chained equations, or applied smoothing with inverse probability weights [44,74]. **Fourth**, our ensemble approach of three base learners offers a conceptually robust strategy that leverages the complementary strengths of different algorithms that can enhance overall performance, even though it did not yield much improvement in our specific case. Di et al. demonstrated that machine learning algorithms are not consistently uniform in their prediction accuracy and model performance and can be highly influenced in context of localized geospatial variations [68]. Therefore, a single fitting model will not be optimal for capturing the complex variability and trends of PM_2.5_ concentrations across all spatiotemporal resolutions.

## 5. Conclusion

To the best of our knowledge, this is one of the first machine learning models to estimate PM_2.5_ concentrations with good model performance and predictive accuracy at the urban level in the Mediterranean region by incorporating a diverse range of publicly available data. We developed an ensemble model informed by three base machine learning algorithms using 61 total variables to predict daily PM_2.5_ concentrations at a 1 km by 1 km resolution for the greater Athens region from 2007 to 2019, achieving good model accuracy in our estimations, with an overall R^2^ of 0.85 and overall RMSE of 4.18 µg/m^3^, demonstrating robust spatial and temporal performance. Our model estimates indicate that this region had moderately high PM_2.5_ concentrations that varied by location and by year. As environmental epidemiology continues to link fine particulate pollution to adverse health impacts, such high resolution historical estimates become essential for reducing exposure measurement error in health effect estimates [95]. Ultimately, our modeling framework provides a ready-to-deploy blueprint for other regions characterized by a paucity of direct air quality monitoring data.

## Supporting information

S1 Fig Map of the study area with all fixed monitoring stations (black triangle), major ports (red anchor), and the visibility station (red airport). The base map data of southern Greece was obtained from OpenStreetMap (OSM), made available under the Open Database License [96].
(PNG)

S2 Fig The annual mean predicted PM_2.5_ concentrations (µg/m^3^) by sector. We calculated the annual mean PM_2.5_ concentrations by municipal sectors of greater Athens (north, south, east, west, central, and Piraeus).
(PNG)

S3 Fig Monthly PM_2.5_ concentrations for each year across greater Athens. (PNG)

S1 Table PM_2.5_ data availability from fixed monitors in the greater Athens area used to calibrate modeled PM_2.5_ in 2007–2019. A total of 11 distinct monitoring stations, with 29,368 particulate matter observations, were recorded from 2007 to 2019. Observations from four original sites were ultimately excluded from the model training and validation due to insufficient data availability.
(DOCX)

S2 Table Predictors used in the base learners (RF, GB, NN). (DOCX)

S3 Table Model input parameters for all base learners and the ensemble model. All programming was implemented in the R software version 4.5.1 using the package “H2O” version 3.46.0.10.
(DOCX)

S4 Table 10-fold cross-validated R2 and RMSE for all three base learners and ensemble models. (DOCX)
