# Supplementary figures and images for: Development of a predictive model for PM2.5 over the greater Athens metropolitan area, Greece, at a 1 km by 1 km grid using satellite measurements and machine learning methods
Source: PLoS One. 2026 Jul 6;21(7):e0352975. doi: 10.1371/journal.pone.0352975 (PMC13336161; doi:10.1371/journal.pone.0352975)

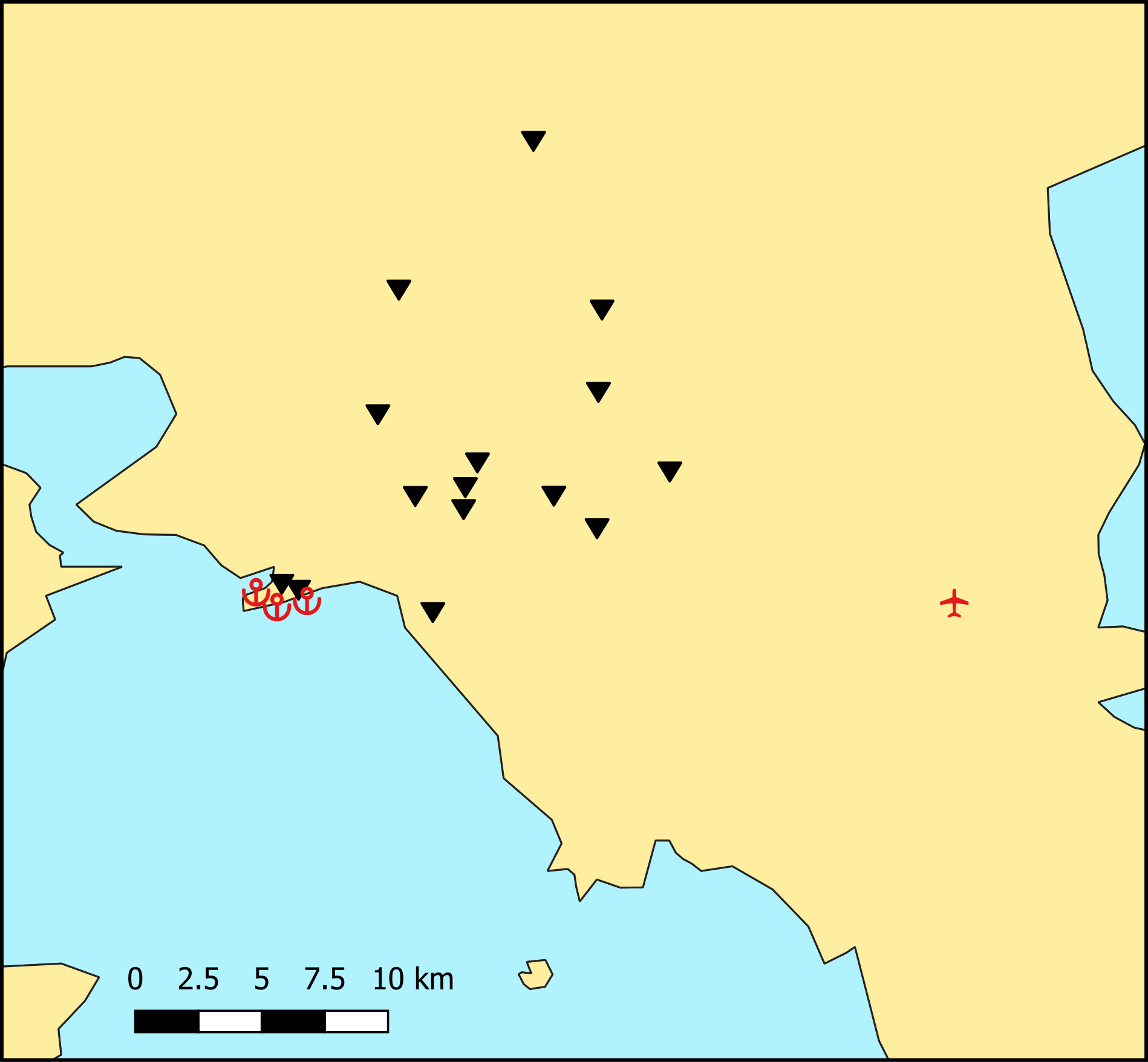

Supplement: S1 Fig — The base map data of southern Greece was obtained from OpenStreetMap (OSM), made available under the Open Database License [96]. (PNG) [file pone.0352975.s001.png]

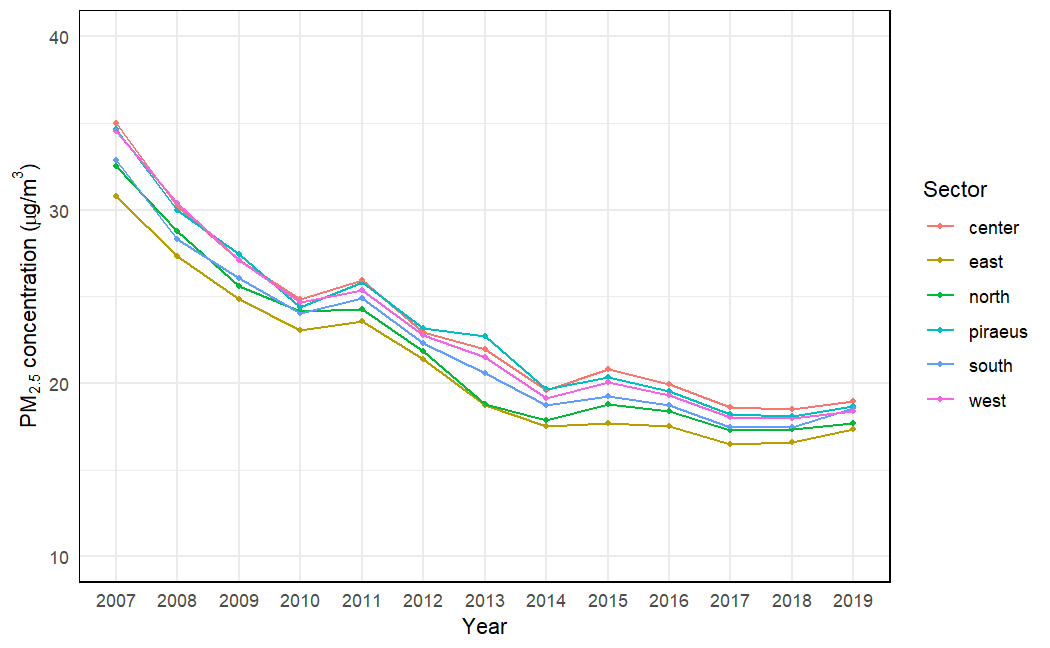

Supplement: S2 Fig — We calculated the annual mean PM2.5 concentrations by municipal sectors of greater Athens (north, south, east, west, central, and Piraeus). (PNG) [file pone.0352975.s002.png]

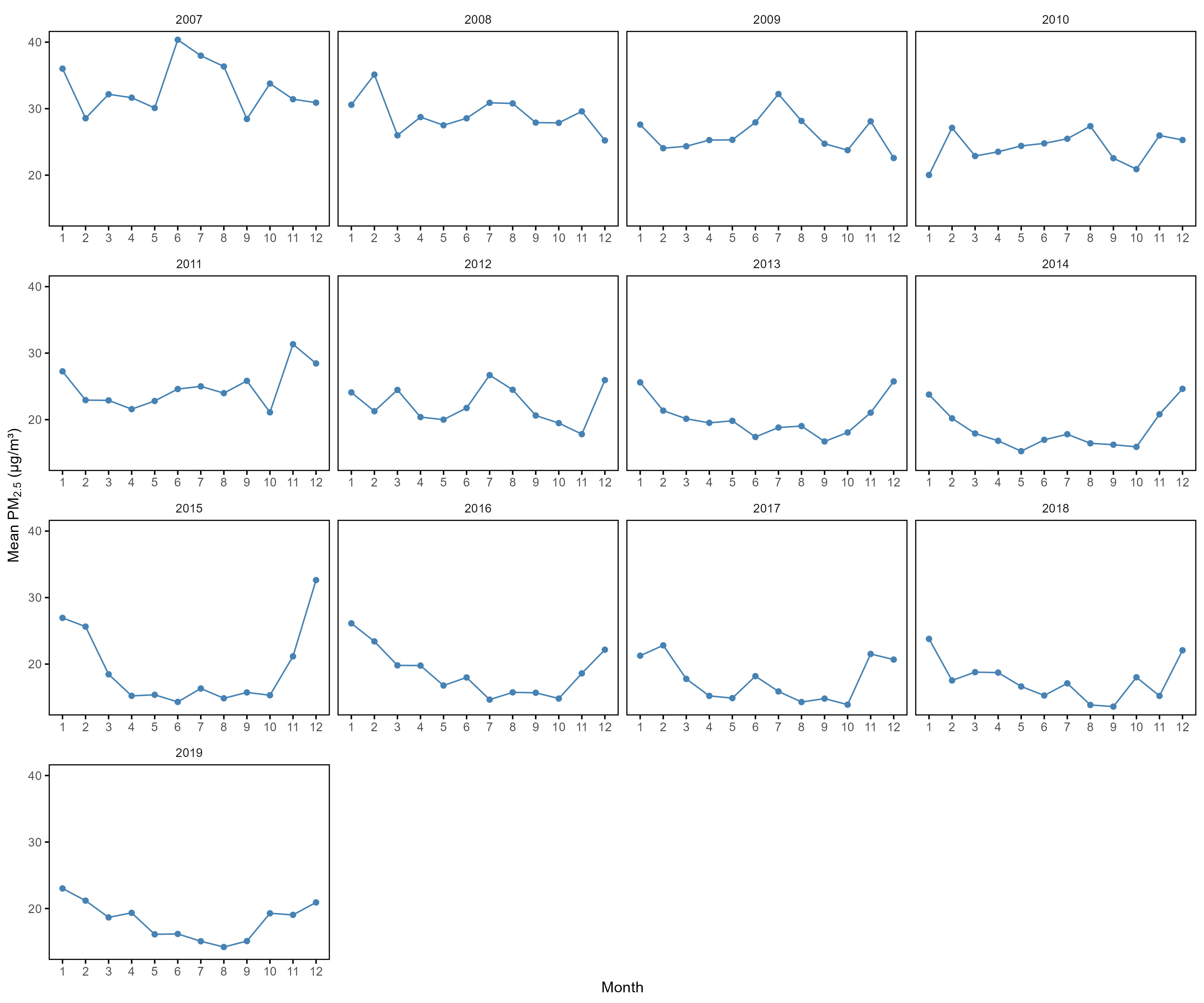

Supplement: S3 Fig — (PNG) [file pone.0352975.s003.png]
