# Supplementary material for: Development of a predictive model for PM2.5 over the greater Athens metropolitan area, Greece, at a 1 km by 1 km grid using satellite measurements and machine learning methods
Source: PLoS One. 2026 Jul 6;21(7):e0352975. doi: 10.1371/journal.pone.0352975 (PMC13336161; doi:10.1371/journal.pone.0352975)
Supplement: S1 Table — A total of 11 distinct monitoring stations, with 29,368 particulate matter observations, were recorded from 2007 to 2019. Observations from four original sites were ultimately excluded from the model training and validation due to insufficient data availability. (DOCX) [file pone.0352975.s004.docx]

| **Sampling site** | **Longitude, Latitude** | **Number of observed**  **samples recorded** | **Number of validated**  **samples added** |
| --- | --- | --- | --- |
| AGP | 23.819, 37.995 | 3741 | 533 |
| ARI | 23.728, 37.988 | 1507 | 2801 |
| GOU | 23.767, 37.984 | 0 | 268 |
| LIO | 23.698, 38.077 | 0 | 1010 |
| LYK | 23.789, 38.068 | 3480 | 873 |
| MAR | 23.787, 38.031 | 0 | 4091 |
| PAN | 23.787, 37.969 | 0 | 351 |
| PER | 23.688, 38.021 | 0 | 1560 |
| PIR | 23.645, 37.945 | 3120 | 842 |
| SMY | 23.713, 37.932 | 0 | 1587 |
| THR | 23.758, 38.144 | 1497 | 2107 |
