# Supplementary material for: Development of a predictive model for PM2.5 over the greater Athens metropolitan area, Greece, at a 1 km by 1 km grid using satellite measurements and machine learning methods
Source: PLoS One. 2026 Jul 6;21(7):e0352975. doi: 10.1371/journal.pone.0352975 (PMC13336161; doi:10.1371/journal.pone.0352975)
Supplement: S2 Table — (DOCX) [file pone.0352975.s005.docx]

| **Predictor** | **Data source** |
| --- | --- |
| **Year** | -- |
| **Month** | -- |
| **Day** | -- |
| **Weekday** | -- |
| **Longitude** | -- |
| **Latitude** | -- |
| **Dust-day indicator** | Central weather monitor provided by the National Observatory of Athens |
| **Mean 24-hour temperature** | Central weather monitor provided by the National Observatory of Athens |
| **Mean relative humidity** | Central weather monitor provided by the National Observatory of Athens |
| **Mean wind speed** | Central weather monitor provided by the National Observatory of Athens |
| **Wind direction by 17 sectors** | Central weather monitor provided by the National Observatory of Athens |
| **NO_2_** | Air pollution monitors provided by GMEE |
| **Elevation** | USGS SRTM |
| **NDVI** | NASA AVHRR |
| **Road density – all roads** | OpenStreetMap |
| **Continuous urban fabric** | CLMS – Urban Atlas |
| **Discontinuous dense urban fabric** | CLMS – Urban Atlas |
| **Discontinuous medium density urban fabric** | CLMS – Urban Atlas |
| **Discontinuous low density urban fabric** | CLMS – Urban Atlas |
| **Discontinuous very low density urban fabric** | CLMS – Urban Atlas |
| **Industrial, commercial, public, military, and private units** | CLMS – Urban Atlas |
| **Railways and associated land** | CLMS – Urban Atlas |
| **Port areas** | CLMS – Urban Atlas |
| **Green urban areas** | CLMS – Urban Atlas |
| **Sports and leisure facilities** | CLMS – Urban Atlas |
| **Forests** | CLMS – Urban Atlas |
| **Herbaceous vegetation associations** | CLMS – Urban Atlas |
| **Mean AOD – 550 nm** | MODIS MAIAC |
| **Mean visibility** | NOAA NCEI ISD |
| **Boundary layer height** | ERA5 |
| **Downward UV radiation** | ERA5 |
| **Evaporation** | ERA5 |
| **Forecast albedo** | ERA5 |
| **Instantaneous 10m wind gust** | ERA5 |
| **Surface pressure** | ERA5 |
| **Total cloud cover** | ERA5 |
| **Total precipitation** | ERA5 |
| **Black carbon surface mass concentration** | MERRA2 |
| **Black carbon column mass density** | MERRA2 |
| **Black carbon u-wind mass flux** | MERRA2 |
| **Black carbon v-wind mass flux** | MERRA2 |
| **Black carbon Angstrom parameter** | MERRA2 |
| **Black carbon extinction AOD** | MERRA2 |
| **Black carbon scattering AOD** | MERRA2 |
| **Organic dust surface mass concentration** | MERRA2 |
| **Organic dust column mass density** | MERRA2 |
| **Organic dust extinction AOD** | MERRA2 |
| **Organic dust scattering AOD** | MERRA2 |
| **SO4 surface mass concentration** | MERRA2 |
| **SO4 column mass density** | MERRA2 |
| **SO4 extinction AOD** | MERRA2 |
| **SO4 scattering AOD** | MERRA2 |
| **Dust surface mass concentration** | MERRA2 |
| **Dust column mass density** | MERRA2 |
| **Dust u-wind mass flux** | MERRA2 |
| **Dust v-wind mass flux** | MERRA2 |
| **Dust Angstrom parameter** | MERRA2 |
| **Dust extinction AOT** | MERRA2 |
| **Dust scattering AOT** | MERRA2 |
| **Total aerosol extinction AOT** | MERRA2 |
| **Total aerosol scattering AOT** | MERRA2 |
