# Supplementary material for: Development of a predictive model for PM2.5 over the greater Athens metropolitan area, Greece, at a 1 km by 1 km grid using satellite measurements and machine learning methods
Source: PLoS One. 2026 Jul 6;21(7):e0352975. doi: 10.1371/journal.pone.0352975 (PMC13336161; doi:10.1371/journal.pone.0352975)
Supplement: S3 Table — All programming was implemented in the R software version 4.5.1 using the package “H2O” version 3.46.0.10. (DOCX) [file pone.0352975.s006.docx]

**Random Forest**

| **Parameter name** | **Final value after testing** |
| --- | --- |
| Number of trees | 1000 |
| Sample rate | 0.85 |
| Maximum tree depth | 35 |
| Number of bins for numerical columns | 1024 |
| Number of bins for categorical columns | 1024 |
| N-folds | 10 |

**Gradient Boosting**

| **Parameter name** | **Final value after testing** |
| --- | --- |
| Number of trees | 1000 |
| Learn rate | 0.1 |
| Maximum tree depth | 8 |
| Number of bins for numerical columns | 1024 |
| Number of bins for categorical columns | 1024 |
| N-folds | 10 |

**Neural Network**

| **Parameter name** | **Final value after testing** |
| --- | --- |
| Epochs | 150 |
| Learn rate | 0.005 |
| N-folds | 10 |

**Ensemble model**

| **Parameter name** | **Final value after testing** |
| --- | --- |
| Base models | DRF, GBM, Neural Network |
| Meta-learner algorithm | GLM with non-negative weights |
| Meta-learner N-folds | 10 |
