# Supplementary material for: Development of a predictive model for PM2.5 over the greater Athens metropolitan area, Greece, at a 1 km by 1 km grid using satellite measurements and machine learning methods
Source: PLoS One. 2026 Jul 6;21(7):e0352975. doi: 10.1371/journal.pone.0352975 (PMC13336161; doi:10.1371/journal.pone.0352975)
Supplement: S4 Table — (DOCX) [file pone.0352975.s007.docx]

|  | **Ensemble**  **(Full data)** | | **Ensemble**  **(Monitor**  **PM_2.5_ values**  **only)** | | **RF** | | **GBM** | | **Neural Network** | |
| --- | --- | --- | --- | --- | --- | --- | --- | --- | --- | --- |
| **Folds** | **R2** | **RMSE** | **R2** | **RMSE** | **R2** | **RMSE** | **R2** | **RMSE** | **R2** | **RMSE** |
| 1 | 0.857 | 4.027 | 0.776 | 4.765 | 0.816 | 4.611 | 0.843 | 4.219 | 0.806 | 4.754 |
| 2 | 0.846 | 4.552 | 0.789 | 5.035 | 0.822 | 4.451 | 0.837 | 4.395 | 0.772 | 4.910 |
| 3 | 0.864 | 4.281 | 0.845 | 4.155 | 0.826 | 4.493 | 0.858 | 4.015 | 0.800 | 5.113 |
| 4 | 0.864 | 3.838 | 0.824 | 4.479 | 0.809 | 5.210 | 0.851 | 4.202 | 0.811 | 4.566 |
| 5 | 0.849 | 4.189 | 0.869 | 4.259 | 0.831 | 4.379 | 0.834 | 4.340 | 0.781 | 5.152 |
| 6 | 0.847 | 4.189 | 0.809 | 4.466 | 0.813 | 4.841 | 0.835 | 4.504 | 0.796 | 4.976 |
| 7 | 0.862 | 4.008 | 0.841 | 3.971 | 0.852 | 4.235 | 0.855 | 4.283 | 0.801 | 5.173 |
| 8 | 0.841 | 4.193 | 0.835 | 4.694 | 0.823 | 4.414 | 0.849 | 4.205 | 0.798 | 4.931 |
| 9 | 0.849 | 4.294 | 0.814 | 4.493 | 0.823 | 4.661 | 0.832 | 4.730 | 0.816 | 4.546 |
| 10 | 0.861 | 4.175 | 0.849 | 4.401 | 0.809 | 4.717 | 0.840 | 4.361 | 0.806 | 4.888 |
| **Mean** | **0.854** | **4.175** | **0.825** | **4.471** | **0.823** | **4.601** | **0.843** | **4.325** | **0.799** | **4.901** |
